# Contaminant driven genetic erosion and associated hypotheses on alleles loss, reduced population growth rate and increased susceptibility to future stressors: an essay

**DOI:** 10.1007/s10646-013-1070-0

**Published:** 2013-04-20

**Authors:** Rui Ribeiro, Isabel Lopes

**Affiliations:** 1 Department of Life Sciences, IMAR-Instituto do Mar, University of Coimbra, Coimbra, Portugal; 2 Department of Biology & CESAM, University of Aveiro, Campus de Santiago, 3810-193 Aveiro, Portugal

**Keywords:** Microevolution, Genetic diversity, Evolutionary ecotoxicology, Resistance tolerance, Inversely sensitive genotype

## Abstract

Microevolution due to pollution can occur mainly through genetic drift bottlenecks, especially of small sized populations facing intense lethal pulses of contaminants, through mutations, increasing allelic diversity, and through natural selection, with the disappearance of the most sensitive genotypes. This loss of genotypes can lead to serious effects if coupled to specific hypothetical scenarios. These may be categorized as leading, first, to the loss of alleles—the recessive tolerance inheritance hypothesis. Second, leading to a reduction of the population growth rate—the mutational load and fitness costs hypotheses. Third, leading to an increased susceptibility of further genetic erosion both at future inputs of the same contaminant—differential physiological recovery, endpoints (dis)association, and differential phenotypic plasticity hypotheses—and at sequential or simultaneous inputs of other contaminants—the multiple stressors differential tolerance hypothesis. Species in narrowly fluctuating environments (tropics and deep sea) may have a particularly high susceptibility to genetic erosion—the *Plus ça change* (*plus c’est la meme chose*) hypothesis. A discussion on the consequences of these hypotheses is what this essay aimed at.

## Introduction

In theory, environmental contaminants can produce microevolutionary changes through the violation of each of the five assumptions of the Hardy–Weinberg theorem: absence of mutations (and the subsidiary assumption on meiotic drive), genetic drift, non-random mating, gene flow, and natural selection. The violation of the subsidiary assumption on sex-linked loci (Falconer and Mackay 1996; Futuyma 2009) can be regarded, at this point, as less pertinent.

First and more obvious, new alleles and new genotypes are expected to be added to the population genetic make up if contaminants are mutagenic at sub-lethal concentrations (Hoffmann and Parsons 1991, 1997; Belfiore and Anderson 2001; De Wolf et al. 2004). Even if not, recombination and mutation rates much often increase with stress (Hoffmann and Parsons 1991, 1997). “[…] recent evidence suggests that during periods of environmental challenge and subsequent stress of the organism, the rates of many mutational events increase dramatically” (Taddei et al. 1997). More frequent mutagenesis in response to stress, through DNA replication and repair infidelity, recombinational DNA rearrangements and the acquisition of genetic information from other organisms (i.e. horizontal transfer), can result from activating mutagenic responses and inhibiting antimutagenic activities (Taddei et al. 1997). Furthermore, frequencies of both mutations and recombinations could be adaptive, being enhanced, at the population level, by contaminant driven directional selection (Hoffmann and Parsons 1991, 1997; Taddei et al. 1997).

Second and also evident, random changes in the population genetic structure are expected in previously small sized populations in the presence of partially lethal concentrations of contaminants. Genetic drift is also expected for larger populations if the contaminant input is intense enough to randomly decimate almost the entire population–contaminant driven bottlenecks. The chances of survival could be dictated by, for instance, a larger distance to the input source or the presence of protective barriers. The scenario where survivors would be the most tolerant individuals cannot be contemplated here, since it would not be genetic drift but, instead, natural selection, which will be tackled ahead.

Third and very much less obvious is the loss of panmixis. Behavioral alterations might dictate changes in mating preference, namely by interfering with chemical signaling and communication. However, aside from the classical assumption of effects caused by direct contaminant interferences on biochemical pathways and physiological processes, there is another plausible possibility for contaminant driven panmixis loss. Although being a largely neglected perspective in ecotoxicity studies, by acting as habitat disruptors and fragmentors, contaminants can also affect population dynamics, community structure and ecosystem functioning with no effects whatsoever at the individual level of biological organization (Dickman et al. 2007). A good example of pollution leading to habitat disruption and, then, to microevolution is the classical case of industrial melanism in the peppered moth (Ridley 2004; Futuyma 2009). Also, a patchy distribution of contaminants could increase the isolation of groups of individuals within the initial population spatial area—demes—and, thus, promote non-random mating with increased homozygosity, even if each deme is in Hardy–Weinberg equilibrium–the Wahlund effect (Ridley 2004). Over time, and as a result of genetic stochasticity, the smaller the deme the higher the homozygosity, by genetic drift and/or inbreeding, and the higher the risk of fixation of alleles, by genetic drift (Bijlsma et al. 1997; Bürger and Lynch 1997; Pigliucci 2001; Ridley 2004; Futuyma 2009).

Fourth, contaminant driven enhanced incursion of alleles and genotypes through immigration is rather difficult to hypothesize, but, and calling also upon the concept of contaminants as habitats disruptors, enhanced emigration seems to be a plausible scenario in real situations of contamination (Amiard-Triquet 2011; Ribeiro et al. 2012). Although the classical and almost exclusive approach of forced exposure in ecotoxicity testing prevented data gathering on spatial avoidance, it is reasonable to assume that at least some organisms may escape contaminated habitats, both through passive emigration—drift—or through active self propelled displacement. Repellence of insecticides is a known example of active avoidance by mosquitoes. At the population level, consequences of emigration match those of mortality (Rosa et al. 2012). If the emigration probability of each individual is set or not by a specific selectable trait, such as its sensitivity to the contaminant, then the outcome on the population genetic structure ends up to be similar to those of natural selection (below) or genetic drift (above), respectively. The emigration of the most sensitive individuals apparently creates a paradox, since it could be regarded as gene flow, but it leads to adaptive evolution. Therefore, it can only be categorized as natural selection and, hereafter, it will be dealt with as such.

Fifth, contaminants can act as a selective pressure, both indirectly by altering interspecific relationship patterns, as increased predation, competition or parasitism, or directly by eliminating the most sensitive genotypes through death or through reduced fitness (differential avoidance included) at partially lethal or at sublethal concentrations, respectively.

The violation of the assumptions of the Hardy–Weinberg theorem corresponds to Bickam’s categories of population genetic impacts (Bickham 2011), with the exception of panmixis loss. Departing from Bickham’s four cornerstones, a geometrical analogy of the null model evolution–a population in Hardy–Weinberg equilibrium–could be a stella octangula. The violation of the first assumption (novel genetic information) would alter its volume, while violations of the later four assumptions would alter the surface of the four faces of each intersecting tetrahedron. Only a polyhedral compound could account for all possible interactions.

The loss of genetic diversity due to pollution—contaminant driven genetic erosion (van Straalen and Timmermans 2002)—could provoke serious short- and long-term negative effects at the population level (Table 1). At least three plausible consequences can be envisaged: the loss of alleles, the reduction of the population growth rate and the increase of the susceptibility of further genetic erosion by future stressors. Expectations on these outcomes under real scenarios of contamination are dependent on the verification of associated non-mutually exclusive hypotheses (Table 1), which discussion is what this essay aimed at. Each of these hypotheses would perhaps merit a separate literature review. However, the purpose here was only to present an overall appraisal on their transgenerational consequences, with illustrating examples. Consequences at larger temporal and spatial scales were here left aside: “a species that an ecologist reports as susceptible to local extinction may be relatively resistant to global extinction. […]. Biologists tend to expect major changes in the physical environment to result either in evolution or extinction. There is, however, much evidence against this generalization” (Sheldon 1997).

**Table 1:** Contaminant driven genetic erosion and associated hypotheses on alleles loss, reduced population growth rate and increased susceptibility to future stressors

Hypothesis	Essence of the argument	Possible transgenerational consequences
Contaminant driven genetic erosion	A contaminant can eliminate genotypes from natural populations	Impoverishment of the gene pool and, thus, loss of genetic diversity
Recessive tolerance inheritance	A contaminant can eliminate alleles if tolerance is a fully or incompletely recessive (or incompletely dominant) trait	Irreversible loss of genetic diversity
Mutational load	A contaminant can cause or induce mutations and, in small populations, average fitness can be reduced due to the accumulation of slightly deleterious mutations	If selection is hard then population downsizing and, possibly, extinction from mutational meltdown
Fitness costs	A contaminant can reduce population average fitness ensuing from physiological (energy re-allocation) and genetic (negative pleiotropy and epistasis) processes during individual tolerance acquisition	Population downsizing and, thus, enhanced susceptibility to future genetic erosion
Differential physiological recovery	Sequential exposures to a contaminant can increasingly eliminate genotypes if tolerant ones are the least able to physiologically recover	Loss of genetic diversity and enhanced susceptibility to future genetic erosion
Endpoints (dis)association	Sequential exposures to different levels of a contaminant can increasingly eliminate genotypes if the ones tolerant to lethal levels are the most sensitive to sublethal levels (or vice versa)	Loss of genetic diversity and enhanced susceptibility to future genetic erosion
Differential phenotypic plasticity	Sequential exposures to different levels of a contaminant can increasingly eliminate genotypes if tolerant ones are the least able to acclimate (through phenotypic plasticity)	Loss of genetic diversity and enhanced susceptibility to future genetic erosion
Multiple stressors differential tolerance	Sequential (or simultaneous) exposures to different contaminants can increasingly eliminate genotypes if the ones tolerant to the first contaminant are the most sensitive to the second	Loss of genetic diversity and enhanced susceptibility to future genetic erosion
*Plus ça change (plus c’est la meme chose)*	Species in narrowly fluctuating environments (tropics and deep sea) have a small among and within genotypes variation	High susceptibility to genetic erosion and, ultimately, population extinction

## The genetic erosion hypothesis

From a pragmatic perspective, the conservation of biological diversity includes preserving (i) habitats, ecosystems and landscapes diversity, (ii) species diversity and (iii) genetic diversity (Dowes 1996; Bickham et al. 2000). This latter refers to the differences among individuals within a population or species that is determined by genotype variation. “Individuals can be compared for many traits and so genetic variation in general is a meaningless term unless it is specified to which trait it refers” (van Straalen and Timmermans 2002). Therefore, genetic erosion is the loss of genotypes determining a specific trait or set of traits. To investigate contaminant driven genetic erosion, two approaches have been commonly used: monitoring selectable markers versus neutral markers (Cousyn et al. 2001; van Straalen and Timmermans 2002; Hoffmann and Willi 2008; Leinonen et al. 2008). The former are genetically determined traits conferring to the individual carrier a fitness advantage or disadvantage under a stressed environment. Selectable traits are, thus, those which proportion in a population is liable to change by a selection pressure, namely a contaminant. Neutral markers are polymorphisms that are not directly affected by the selective agent. Genetic erosion in contaminant driven bottlenecked populations is expected to be adequately quantified with neutral markers, unless mutagenicity and genotoxicity of the chemicals give birth to new alleles and new alleles’ combinations (Nadig et al. 1998; Pfrender et al. 2000; Belfiore and Anderson 2001; van Straalen and Timmermans 2002; Matson et al. 2006; Hoffmann and Willi 2008; Nowak et al. 2009; Soeter et al. 2010).

Already in 2002, van Straalen and Timmermans wrote “Our review has singled out several studies showing that environmental stress may significantly reduce the genetic variation in a population. In this sense the genetic erosion hypothesis is confirmed”. Later works reinforced this claim for a wide range of taxonomic groups, exhibiting different modes of reproduction and inhabiting both terrestrial and aquatic (salt- and freshwater) environments (e.g. Keane et al. 2005; Ward and Robinson 2005; Gardeström et al. 2008; Nowak et al. 2009; Ribeiro et al. 2012).

## The recessive tolerance inheritance hypothesis

Due to its possible irreversibility, contaminant driven loss of alleles is of utmost concern. This would be straightforward if tolerance, in non-haploid species, was a homozygous fully or incompletely recessive trait. The dominant alleles conferring non-tolerance would be lost quickly and their recuperation would depend on immigration or reverse mutation. On the other extreme of this worst-case scenario, tolerance inheritance by over- or underdominance—heterozygotes being more or less tolerant than either homozygote, respectively—would guarantee the presence of all alleles. In sexually reproducing species, lost genotypes regarding tolerance would arise in the very next generation. Heterozygosity tends to correlate with fitness, especially under stressful conditions (Parsons 1997), making overdominance a likely hypothesis. This could be so due to the higher metabolic efficiency, with consequently lower energy requirements, that heterozygotes tend to exhibit relatively to homozygotes (Parsons 1997). Three other tolerance inheritance alternatives exist: dominance, incomplete dominance and no dominance. The former would lead to the elimination of homozygous recessive sensitive genotypes, but their presence in subsequent generations would be assured by heterozygous crossing. In the latter alternatives, if the contaminant pulse was strong enough to fully eliminate sensitive and intermediately tolerant genotypes, then the outcome would be the worst possible: allele fixation. If contamination was weak enough to eliminate only the sensitive homozygous genotypes, then the outcome would match that of the dominance alternative. Indeed, the degree of dominance can change depending on the contaminant concentration (Macnair 1997): “If the dose–response curve of the heterozygote is intermediate between those of the two homozygotes, then it will follow that at low testing concentrations tolerance will be dominant, while at high concentrations it will be recessive”. Furthermore, the genetic background in which the gene is found can also influence the dominance, with, for instance, modifier genes rather than the major gene, determining dose–response curves (Macnair 1997).

Tolerance as a polymorphism is common in pesticide-targeted organisms and classical examples include the incomplete dominance of mosquitoes tolerance to permethrin, controlled by a single gene (Ridley 2004). Tolerance was found to be completely or partially dominant in several studies (Hoffmann and Parsons 1991, 1997; Macnair 1997), which was expected because alleles expressed in heterozygotes will evolve more rapidly than recessive ones (Macnair 1997). Genetically determined tolerance to contaminants can be a continuous variable (e.g. Lopes et al. 2004a). Such continuity often ensues from polygeny, complicating inheritance (Hill 2010). Moreover, the same genes frequently do not influence variation in a trait across all stress levels, i.e. the intensity of selection influences the number of loci involved in the response to a stressor (Hoffmann and Parsons 1991, 1997; Macnair 1997). Under mild directional selection, tolerance tends to have a polygenic basis, with many minor genes, while under intense hard directional selection, major genes tend to be favored (Hoffmann and Parsons 1991, 1997; Macnair 1997). For example, DDT tolerance by insects tended to be a polygenic response when the pressure killed only 80 to 90 % of laboratory populations, while a monogenic tolerance is expected in the much larger field populations exposed to pest control (deliberately fully lethal) doses (reviewed by Hoffmann and Parsons 1991).

## The mutational load hypothesis

Besides leading to a higher probability of alleles’ fixation, population downsizing may also end up in local extinction if due to a reduced population growth. Not being mutually exclusive, two pathways resulting in such a poorer average fitness are conceivable: mutational load and tolerance associated fitness costs.

The purely neutral theory of evolution predicts that species with larger populations would have more genetic variation, which is not empirically confirmed; variation being much the same irrespectively of population sizes (Ridley 2004). Instead, if most mutations are nearly, rather than exactly, neutral, being slightly disadvantageous, as postulated by the nearly neutral theory, then they would be eliminated in species with large populations, due to the prevalence of selection over genetic drift, or be ignored, behaving as effectively neutral, in species with small populations, where selection is weak relative to random drift (Ridley 2004). In the latter populations, slightly deleterious mutations may drift up in frequency, contributing to the observed genetic variation (Ridley 2004). The individual carriers of the deleterious allele will suffer “genetic death” by their failure to survive or to reproduce, thereby reducing the population average fitness (Falconer and Mackay 1996). Since most species produce more offspring than the habitat resources can support, selection will be soft with genetically dead individuals being replaced by competitors, with populations maintaining their densities at the carrying capacity (Falconer and Mackay 1996). There are examples of an intense (up to 50 %) mutational load–proportion of the population that suffers genetic death, i.e. the proportional reduction of population average fitness due to the deleterious allele occurrence–being present in flourishing populations (Falconer and Mackay 1996). However, if the load is intense enough to disallow individuals to replace themselves, then the population size declines in a self-accelerating process–the extinction vortex (Bürger and Lynch 1997; Höglund 2009). The gradual accumulation of mildly deleterious mutations leading to a decreased population average fitness and to the eventual rapid local extinction—mutational meltdown—has not only been shown in asexual populations, but it is also potentially very relevant in sexual species with small (below one hundred individuals) populations (Bürger and Lynch 1997; Höglund 2009). Furthermore, mutational load was also shown to pose a significant long-term risk of extinction to even moderately large sexual populations (Bürger and Lynch 1997; Höglund 2009).

## The fitness costs hypothesis

The acquisition of tolerance to chemical stress may involve fitness costs, ensuing from physiological, through energy re-allocation from fitness enhancing functions to detoxification mechanisms, and genetic processes, through antagonistic pleiotropy and epistasis (Hoffmann and Parsons 1991, Shirley and Sibley 1999; Klerks et al. 2011). However, examples in the literature report not only the presence, but also the absence of such fitness costs (Arnaud and Haubruge 2002; Lopes et al. 2004a; Liu and Han 2006; Fisker et al. 2011; Mouneyrac et al. 2011; Kilot and Ghanim 2012; Saro et al. 2012; Ribeiro et al. 2012). This absence is expected if the selective pressure targets energy carriers, favouring metabolic efficiency, justifying the predominance of studies reporting positive correlations among life history traits and tolerance (Parsons 1997).

## The differential physiological recovery hypothesis

The repeated application of a pesticide in a crop area or inputs of metals rich acid mine drainage when it rains after prolonged droughts are examples of sequential contaminant pulses. The physiological recovery rate after a contaminant pulse may vary among individuals of the exposed population. If this rate is mechanistically linked to tolerance, then the most tolerant genotypes would present a double advantage. Such a linkage is expected if physiological processes determining tolerance, for instance detoxification pathways, are the same determining a faster physiological recovery. If the time lag between two sequential partially lethal pulses of the same contaminant at similar concentrations is small enough to prevent physiological recovery of all individuals, then the first pulse would eliminate the most sensitive genotypes, while the second pulse would affect those among the tolerant genotypes with the longest recoveries. If physiological recovery and tolerance are positively correlated, then only the very tolerant genotypes will remain, by recovering faster than the intermediately tolerant ones. Therefore, enhanced genetic erosion would be expected with a second pulse. If genotypic variation in physiological recovery exists, but with its rate being determined by mechanisms other than those determining tolerance, then genetic erosion would be even more noticeable. For instance, the speed of internal reserves and energy re-stocking could dictate the physiological recovery time, thus explaining a possible lack of a physiological recovery versus tolerance coupling. Here, the second pulse would eliminate the tolerant genotypes presenting a slower physiological recovery. If tolerance is energetically expensive, then a negative correlation between physiological recovery and tolerance would be probable and the second pulse could genetically erode the population to a much larger extent.

Vis-à-vis the probable high magnitude of intermittent exposures, data on genetic variability and variation in physiological recovery time and its relation with tolerance may be pertinent. Although a substantial amount of studies on intermittent exposure scenarios have already been performed, consequences on populations’ genetic structure merits to be further addressed.

## The endpoints (dis)association hypothesis

The scenario of a prolonged exposure to sublethal levels of a contaminant after an intense lethal pulse is conceivably realistic, namely because pollutants can stay partially bioavailable (e.g. adsorbed to the soil or sediment). As with physiological recovery, if at least some tolerant genotypes remaining in a genetically eroded population after a partially lethal pulse of a stressor are those more sensitive to or less able to cope with, by acclimation and phenotypic plasticity, a latter long-term sublethal exposure to the same stressor, then an enhanced genetic erosion is possible.

The endpoints association hypothesis postulates that tolerant genotypes to lethal levels of a contaminant are also tolerant to sublethal levels. This might be so, namely, if mechanisms conferring tolerance to both levels are the same (e.g. metalothionein detoxification) (Saro et al. 2012). However, lethal responses likely involve specific mechanisms, regulated by a few genes of large effects, while sublethal responses likely involve other, generalist, mechanisms regulated by many genes of small effects (Barata et al. 2000).

Corroborating the endpoints association hypothesis, genetically determined median clonal effective concentrations eliciting active spatial avoidance (AC_50_) and median clonal lethal concentrations (LC_50_) of 12 clonal lineages of the freshwater microcrustacean cladoceran *Daphnia longispina* were highly correlated (AC_50,12h_ versus LC_50,48h_: *r* = 0.57, *p* < 0.05, *n* = 12; Lopes et al. 2004b). Two pulses of copper, one sudden and intense and the other slower and weaker, would eliminate the most sensitive genotypes, by being killed or emigrating, irrespectively of their temporal sequence. However in the latter work on avoidance and lethality, two out of twelve genotypes (I1 and I2 sensu Lopes et al. 2004b) were found to be sensitive to lethal levels but not much able to escape, meaning that they would not evade a first slow exposure but they would die with a second more intense pulse; genetic erosion would then be enhanced. This case illustrates well that, even if positively correlated over the whole population, the occurrence of negative linkages between lethal and sublethal endpoints in some genotypes could augment genetic erosion. In the absence of a positive association, more negative linkages will exacerbate this potential enhancement.

## The differential phenotypic plasticity hypothesis

To the best of our knowledge, the differential acclimation hypothesis was only explicitly addressed by Saro et al. (2012). As these authors put it, acclimation refers to reversible compensatory changes, within hours, days or weeks, during the lifespan of the organism, and phenotypic plasticity is the expression of alternative phenotypes from the same genotype “operating on a slightly longer timescale and more permanent than the acclimatory responses” (Willmer et al. 2000). Nevertheless, in the context of microevolution, acclimation could be regarded as a phenotypic plasticity response (Pigliucci 2001), accepting that this latter also “includes rapidly reversible changes in morphology, physiology, and behaviour” (Futuyma 2009). Since all organism’s features, with the exception of DNA sequences, may be called phenotypes (Futuyma 2009), then developmental conversion, maternal effects, including cross generational acclimation, and epigenetic inheritance, based on genomic imprinting, could also be included in a general so-called differential phenotypic plasticity hypothesis. Looking at temporal trends in the effects of a long-term exposure to sublethal acid mine drainage dilutions on the reproduction of three clonal lineages of *D. longispina* differing in their tolerance to lethal levels of this effluent, Saro et al. (2012) failed to contribute to the verification of this hypothesis, because no evidences of acclimation during the organism lifespan were found. In a larger temporal scale, environmental stress may augment expressed genetic variability, which would normally be suppressed by self-regulatory processes, i.e. canalized (Hoffmann and Parsons 1991, 1997).

## The multiple stressors differential tolerance hypothesis

Genetic diversity is the basis for the adaptation of populations through natural selection and its reduction may compromise population resilience and increase their extinction risk under the occurrence of environmental changes (Maltby et al. 2001). However, at least in ecotoxicology, no much recent evidence altered the opinion that “loss of genetic variation introduces a decrease of adaptive potential […] also holds remains an open question” (van Straalen and Timmermans 2002). Such a decrease is expected to be particularly severe for populations already under genetic (intrinsic) stress, namely small sized populations with excessive homozygosity due to genetic drift and/or inbreeding (Bijlsma et al. 1997; Bürger and Lynch 1997). Sequential inputs of different contaminants are a common situation. An already genetically eroded population may be at a high risk of further genetic erosion if at least some of the remaining genotypes are sensitive to a future stressor (Ribeiro et al. 2012). To predict the potential of a contaminant to provoke genetic erosion and, then, to address the genetic erosion enhancement by a second contaminant, it would help to revisit the genetic erosion hypothesis, looking at its assumptions and refining the meaning of sensitive and tolerant genotypes.

A contaminant will only provoke genetic erosion by directional selection if two assumptions are met at the population level. First, among genotypes variation needs to be high enough to assure that differential genotype susceptibility is present, i.e. both genetically determined sensitive and tolerant genotypes exist. Natural selection can only occur if there is a proportion of genetic variation in the phenotypes of the exposed population—heritability. When facing an increase in contaminant environmental concentrations, a low degree of genetic variation will more easily determine the elimination of the entire set of genotypes, extinguishing the population, while a high degree of genetic variation will prevent the tolerant genotypes to disappear. This latter situation is only apparently a safer one because of the higher probability of using individuals, in toxicity testing, which are not representative of the natural populations’ and species sensitivity. Lack of representativeness may lead to severe under- or overestimation of risk. Classical ecotoxicity testing limited data gathering on intraspecific variability because of the long lasting quest for precision at the expense of accuracy, through the minimization of genetic variation in many tests (Forbes and Calow 1997). This paradigm has pervaded toxicity testing, namely with daphnids where the use of clonal lineages has become the standard.

A second assumption underlying the genetic erosion hypothesis is that within genotypes variation in sensitivity is low enough—high heritability—to prevent that at least a few individuals belonging to each of the sensitive genotypes will remain in the population, even when contaminant concentrations exceed by far their median effective concentrations (EC_50_). A sensitive genotype, i.e. with a significantly lower median effective contaminant concentration, will only be wiped out if almost all of its individuals perish, stop reproducing or emigrate. This reasoning demands for the concept of “critically sensitive” genotypes, which are those meeting both criteria: being sensitive and with a low (non-genetic) variation. This was the case in an experiment carried out with artificial laboratory populations of *D. longispina* comprising five clonal lineages, which differed in their tolerance to lethal levels of a metal rich AMD (Lopes et al. 2009). The two most sensitive lineages were fully eliminated by an intense pulse of AMD.

Summarizing, the two above mentioned assumptions are fulfilled if at least some critically sensitive genotypes occur in natural populations. The genetic erosion potential of a contaminant at a given intensity, e.g. the environmental concentration, could, thus, be expressed as the proportion of critically sensitive genotypes.

When exposed to two contaminants, genotypes critically sensitive to both will be eliminated by the first one, no matter the temporal sequence. Therefore, genetic erosion will not be enhanced by the second stressor. To evaluate the potential enhancement, one should look at the inversely sensitive genotypes: those critically sensitive to one stressor but not to the other. Genotypes critically sensitive to none of the contaminants are safely co-tolerant and will remain in the population. The label “co-tolerant” encompasses here both the concepts of co-tolerance and of multiple tolerance (sensu Macnair 1997). If a positive genetic correlation exists between the tolerance to the two stressors, the enhanced erosion by the second stressor will probably be minimal. A discussion on the mechanisms behind co-tolerance, including genetic linkage and pleiotropy, was beyond this essay aim. Several studies reported such co-tolerance in algae (Knauer et al. 2010), plants (Eränen 2008), bacteria (Tennstedt et al. 2003), oligochaetes (Klerks and Levinton 1989; Reinecke et al. 1999), cladocerans (Lopes et al. 2005; Agra et al. 2010), isopods (Brown 1976), insects (Nowak et al. 2009), and fish (Murdoch and Hebert 1994; Nacci et al. 2002).

### The *D. longispina* example: introduction

The association between the tolerance to lethal levels of several pairs of chemicals by laboratory reared clonal lineages of *D*. *longispina* originating from the aquatic system of an abandoned mine site was evaluated by Lopes et al. (2005). Genetic erosion was shown to occur in the water reservoir receiving AMD produced at the abandoned mine tailings (reviewed by Ribeiro et al. 2012). Lopes et al. (2005) reported only one significant correlation out of six pairwise combinations. Possible generalist versus chemical specific physiological mechanisms responsible for tolerance were discussed, but an analysis of the genetic erosion potential of the chemicals combinations falls outside the scope of this paper. However, their raw data could be revisited.

### The *D. longispina* example: materials and methods

Twelve clonal lineages of *D. longispina* differing in their genetically determined sensitivity to lethal levels of copper were selected by Lopes et al. (2005). To remove environmental and maternal influences on tolerance, each cloned lineage was maintained in the laboratory under controlled optimal conditions for at least 20 generations. Each clonal lineage was exposed to a gradient of nine concentrations of copper, zinc, cadmium, and of the pyrethroid insecticide deltamethrin, plus controls. Five 4 days old juveniles from the third brood were exposed for 48 h in 42 ml glass vessels filled with 40 ml of test solution (renewed at 24 h), with four replicates per test concentration. Mortality was checked after 24 and 48 h of exposure.

With Lopes et al. (2005) raw data, median, lower and upper quartile lethal concentrations (LC_50,48h_, LC_25,48h_ and LC_75,48h_, respectively) were calculated for each clonal lineage using the software Priprobit (Sakuma 1998). For each chemical, phenotypic variance (V_P_) was computed adding the environmental variance (V_E_) and the genetic variance (V_G_), V_E_ being determined with approximate estimates of standard deviation values from the models fitted to mortality versus concentration and approximate values of broad sense heritability for each chemical being determined as V_G_/V_P_ (Falconer and Mackay 1996; Hoffman and Parsons 1997). In the present study, V_E_ only means the non genetically determined component of variance, since differences of environmental conditions among individuals were, at most, minimal, with almost all observed within genotypes variation being a result of stochasticity (Falconer and Mackay 1996; Forbes and Calow 1997). For comparative purposes among chemicals, the genetic variation was quantified with the coefficients of variation (CV) of the median lethal concentrations at 48 h.

Also for comparative purposes, the response variation of each genotype was evaluated with its relative spread of sensitivity, which is the difference between the lower and the upper quartiles relatively to the median effective concentration, i.e. (LC_75,48h_-LC_25,48h_)/LC_50,48h_, in percentage. For each chemical, a clonal lineage was categorized as critically sensitive if its LC_75,48h_ was similar to or below the average of the set of LC_50,48h_ for the twelve clonal lineages. For each pair of chemicals, inversely sensitive clonal lineages were those critically sensitive to one of the chemicals but not to the other. Therefore, safely co-tolerant clonal lineages were those neither critically co-sensitive nor inversely sensitive.

### The *D. longispina* example: results

**Fig. 1:**
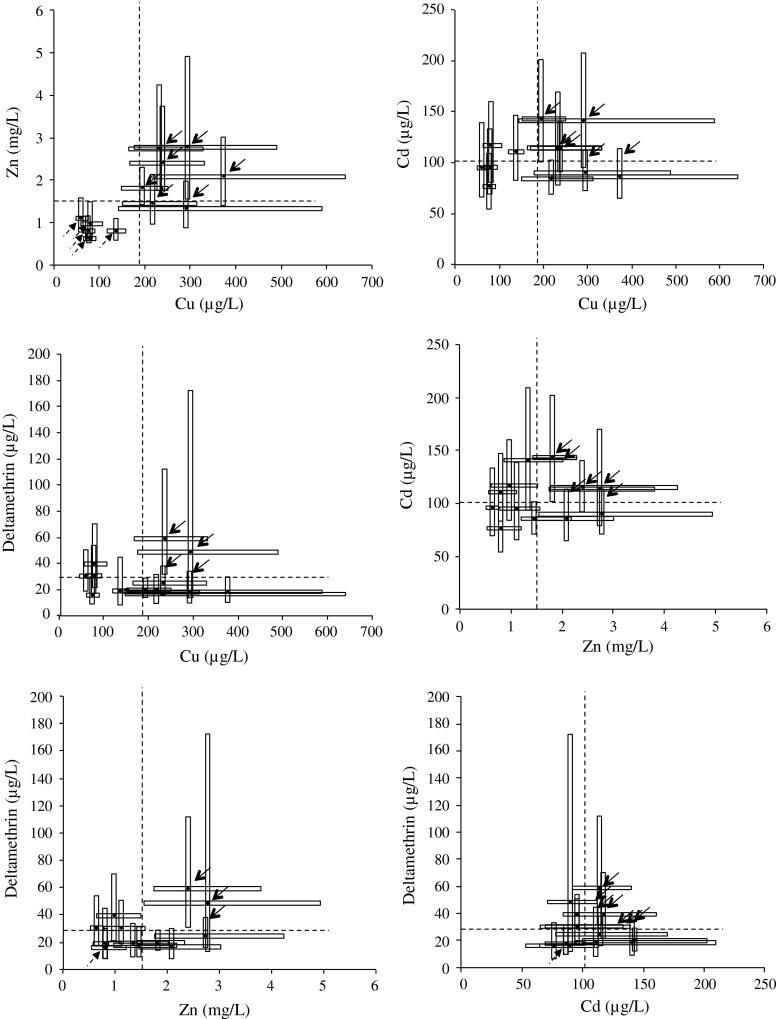
Boxplots (*lower quartile, median and upper quartile*, i.e. LC_25_, LC_50_ and LC_75_, respectively) for the twelve clonal lineages of *Dahnia longispina* and for each pair of the four tested chemicals (copper, zinc, cadmium, and deltamethrin), after 48 h exposures. *Dashed lines* indicate the mean of the set of LC_50,48h_ for the eight clonal lineages. *Solid arrows* indicate the safely co-tolerant clonal lineages and *dashed arrows* indicate the critically co-sensitive lineages for each pair of chemicals

The smallest among genotypes variation in the tolerance to lethal levels after a 48 h exposure was found for cadmium (CV of 20 %), while for copper, zinc and deltamethrin the variation was larger (CV values of 49, 55 and 50 %, respectively) (Fig. 1). The within genotypes variation, measured as the mean of relative spreads of tolerance (observed range inside brackets), was 70 (28 to 153 %), 78 (64 to 121 %), 63 (38 to 81 %), and 143 % (79 to 327 %), for copper, zinc, cadmium, and deltamethrin respectively; with five, five, two, and three out of 12 clonal lineages being critically sensitive to each of these chemicals, respectively. One clonal lineage (E83 sensu Lopes et al. 2005) was found to be critically sensitive to the three tested metals. Sensitivity to lethal concentrations of the pesticide deltamethrin presented the smallest genetic component, with an approximate estimate of broad sense heritability of 18 %, while from two- to threefold higher values were found for the tested metals (58, 63 and 40 %, for copper, zinc and cadmium, respectively).

The only correlated pair of chemicals (Cu|Zn; *r* = 0.73; *p* < 0.01) presented seven safely co-tolerant and no inversely sensitive clonal lineages (Fig. 1). For the other tested pairs, even without negative correlations (*r* ≥ −0.14), the number of inversely sensitive clonal lineages varied between four and nine out of 12; Zn|deltamethrin being the worst-case scenario for paired exposures, with only three safely co-tolerant clonal lineages.

### The *D. longispina* example: discussion

If the 12 clonal lineages were representative of a natural reference population, then the low genetic variation in the tolerance to cadmium (CV of 20 %) would indicate a small difference between partially lethal and fully lethal concentrations, at the population level, and, thus, a small increase in environmental concentrations would more easily determine the elimination of all genotypes. Similarly, such an increase would wipe out the genotypes with low relative spreads of sensitivity for each chemical, like the 28 % value (with copper).

Copper and zinc, with the largest number of critically sensitive genotypes—five out of 12, i.e. 42 %—were the chemicals posing the highest relative risk of genetic erosion at an intermediately lethal concentration (the mean of the set of LC_50,48h_), assuming that clonal lineages here used were representative of natural reference populations. This assumption was far from being fulfilled because the clonal lineages were established with the aim of maximizing the genetic variation in the tolerance to lethal levels of copper. The clonal lineage found to be critically sensitive to all tested metals would always be at a high risk and, therefore, the sequential exposure to intermediately lethal levels of two metals would not change much the risk of its most probable elimination. It is the number of inversely sensitive genotypes that expresses the increased susceptibility to future stressors. Considering the potential for genetic erosion due to an intermediately lethal pulse of each tested chemical and assuming that all clonal lineages critically sensitive to each chemical would be killed, it would be expected that 17 (cadmium) up to 42 % (copper and zinc) of the clonal lineages could be eliminated. However, the loss of genotypes could almost double, attaining 75 %, if a pulse of deltamethrin was followed or preceded by a pulse of zinc, both at intermediately lethal concentrations. This reasoning emphasizes the usefulness of the safely co-tolerant genotype concept, to discuss the potential of contaminant driven genetic erosion by directional selective pressures.

## The *Plus ça change* hypothesis

The *Plus ça change* hypothesis is merely the expansion of the palaeontologist P. R. Sheldon’s *Plus ça change* (*plus c’est la même chose*) model (Sheldon 1996) to shorter temporal timescales, from macro- to microevolution. Within the fossil record, three dominant modes of evolution were found—stasis and occasional punctuations (punctuated equilibrium), directional (gradualism) and random walks (Grey et al. 2012). Linking these evolutionary trends with paleoenvironmental conditions is the core of Sheldon’s model, which predicts that “continuous phyletic evolution is characteristic of relatively stable environments, whereas stasis tends to prevail in unstable environments. […]. In the simplistic terms of this model, the tropics and deeper sea tend to be dominated by long-term (albeit continuously evolving) specialists and shallow marine shelves [and temperate zones] by long-term generalists” (Sheldon 1997).

Despite this model being bound to hard-part morphologies and geological timescales, also species and populations’ sensitivity thresholds in narrowly fluctuating environments could more easily be exceeded (Parsons 1997), by, for instance, chemical pollution, “like a loud sneeze in a hushed library compared with in a football crowd” (Sheldon 1997). Here, abiotic conditions are expected to act in a stabilising mode of selection, which could result in a reduction of among and within genotypes variation in sensitivity to contaminants, enhancing the susceptibility to genetic erosion and, ultimately, population extinction. If so then biological diversity in the tropics and deep sea would be even more at risk than previously believed. Small amplitudes of within population genetic variation would contribute to the anticipated differences in susceptibility among ecosystems across latitudinal gradients: “Specifically, we hypothesize that the effects of anthropogenic disturbance will be greater on stable ecosystems, compared to naturally variable systems. […] We also would expect that ecosystems in tropical environments would be more susceptible to anthropogenic stressors than would their temperate counterparts” (Clements et al. 2001).

## Final remarks

The genetic erosion hypothesis is demonstrated but some of the associated hypotheses here discussed are far from being sufficiently tested. Several studies were carried out, as explicitly suggested by Hoffmann and Parsons (1997), with isofemale strains, such as clonal lineages of cladocerans, which still holds, in our opinion, a great promise. A decade ago, van Straalen and Timmermans (2002) wrote “At the moment, there does not seem to be a sound scientific basis for incorporating genetic diversity measurements into risk assessment”, emphasising the need of further research. We still endorse this belief, avoiding hasty recommendations. This perspective could, however, soon change, if new data would suggest that the situation is more serious than expected, especially regarding the multiple stressors differential tolerance hypothesis. Furthermore, unless strong antagonistic effects occur, the extent of genotype loss after sequential pulses of two contaminants will be similar to or worse than if only one pulse of their mixture takes place. This is so because all genotypes critically sensitive to at least one of the contaminants will be eliminated, with the assumption of antagonism absence. This logical deduction–the theorem of enhanced genetic erosion by multiple simultaneous stressors–merits also to be experimentally considered when evaluating the toxicity of mixtures; “stressors rarely occur in isolation” (Luoma et al. 2001).
